# Comparative Evaluation on the Effect of Different Remineralizing Agents on Enamel-Bracket Shear Bond Strength: An In Vitro Study

**DOI:** 10.7759/cureus.44795

**Published:** 2023-09-06

**Authors:** Suyash S Joshi, Nupur S Ninawe, Naveen Reddy Banda, Urvi Gala, Avani Doiphode, Nilam Honaje

**Affiliations:** 1 Department of Pediatric and Preventive Dentistry, Government Dental College and Hospital, Nagpur, IND; 2 Department of Pediatric and Preventive Dentistry, Ibn Sina National College for Medical Studies, Jeddah, SAU

**Keywords:** 5% sodium fluoride, silver diamine fluoride, cpp-acp paste, shear bond strength, orthodontic brackets

## Abstract

Aim: This study aimed to compare the effects of applying various remineralizing agents before and after acid etching on the enamel-bracket shear bond strength (SBS) in vitro. These agents included silver diamine fluoride (SDF), casein phosphopeptide-amorphous calcium phosphate (CPP-ACP), and 5% sodium fluoride (5% NaF).

Materials and methods: All the selected teeth were divided equally into six subgroups depending on before and after acid etching and one separate control group for the in vitro study design. Eighty-four extracted premolar teeth (12 teeth in each group x seven groups, including the control group). Before acid etching, teeth in groups A1, B1, and C1 were given SDF, CPP-ACP paste, and 5% NaF, respectively. Following acid etching, all of the teeth in Groups A2, B2, and C2 received the same preventative treatments. After that, the SBS of the bonded brackets to the enamel was evaluated.

Results: The CPP-ACP group, control group, and SDF group had the highest values for SBS prior to acid etching.The 5% NaF group had the weakest bonds, and the difference between the groups was statistically significant. The CPP-ACP group had the highest SBS following acid etching, followed by the 5% NaF group. The least bond strength was seen in the SDF group, and the difference between the three groups was significant.

Conclusion: When it comes to bonding orthodontic brackets, the CPP-ACP pretreatment is superior to fluoride pretreatment in terms of effectiveness. The use of these remineralizing agents resulted in favorable values that did not have any effect on the SBS and were therefore safe to use with orthodontic brackets.

## Introduction

Orthodontic treatment, especially that given to children, is intended to enhance dental health, bite, and appearance. However, there are certain potential negative outcomes associated with orthodontic treatment with fixed equipment. Patients with a historical pattern of inadequate dental care are more likely to have these negative outcomes. The formation of white spot lesions (WSLs) surrounding the brackets during orthodontic treatment is still an issue, despite the breakthroughs in orthodontic treatments in recent years [1]. Plaque and food debris have a greater chance of sticking to the flat surfaces of orthodontic bands and brackets, which may lead to tooth cavities. Subsurface enamel porosity caused by demineralization caused by caries leads to WSLs, which look like milky white spots on smooth surfaces. The term subsurface enamel porosity describes this defect [2,3].

The formation of WSLs around orthodontic attachments can begin as early as four weeks into the treatment process [3]. The presence of acidic dental materials in the bracket bonding also increases the chances of the development of caries and the use of fluoride when applied prior to the treatment procedure can reduce the iatrogenic enamel damage [4]. In addition to these measures, remineralizing agents should be used before and throughout orthodontic treatment to ensure optimal results. This is particularly true for those whose enamel is demineralized before therapy even begins. When fluoride or casein phosphopeptide amorphous calcium phosphates (CPP-ACP) were used as a remineralizing agent prior to acid etching, a reduction in bond strength was found [2]. However, some investigations have shown no evidence that remineralizing chemicals weaken bracket bonds [5]. The number of WSLs in orthodontic patients may be decreased using fluoride-containing toothpaste and fluoride-containing mouthwash, as has been shown. Fluoride may also promote the remineralization of WSLs if there is an abundance of calcium and phosphorus in the plaque or saliva. For every one fluoride ion, 10 calcium ions, six phosphate ions, and two fluoride ions are needed to make a single unit cell of fluorapatite (Ca10(PO4)6 F2) [6]. The presence of acidic dental materials in the bracket bonding also increases the chances of the development of caries, and the use of fluoride when applied prior to the treatment procedure can reduce the iatrogenic enamel damage [7].

Enamel surface treatment with fluoride varnish has the potential to weaken the bond strength of bonded brackets, which could ultimately result in bracket failure. There are a few different ways that topical fluoride might be applied during the bonding process for orthodontic brackets. Fluoride application strategies before acid etching, during acid etching when the fluoride is introduced to the acid etchant, and after acid etching on the bond strength of orthodontic brackets have been studied before, with conflicting findings [8,9]. A few of the studies have also pointed out that when fluoride varnish was added either before or after acid etching, the results of various studies demonstrated a considerable decrease in the bond strength values [10]. Meanwhile, other research has shown that applying fluoride varnish to enamel surfaces before or after acid etching has no effect on the strength of the bracket bond [11].

Silver diamine fluoride (SDF), a clear liquid that combines the antibacterial effects of silver with the remineralizing effects of fluoride, could be used to treat carious lesions in babies and people with special health care needs [12]. By moderating the impacts that bacteria have on the hard tissue while simultaneously increasing remineralization, SDF becomes one of the strategies that can be used as a remineralizing agent to combat caries. This is one of the most effective cariostatic topical agents and one that is not only affordable but also easily accessible. SDF is capable of storing fluoride in the subsurface at a rate that is two to three times higher than any other fluoride solution [12]. Apart from the above-mentioned components, recent studies have also used bioactive glass for dental prophylaxis and remineralization after orthodontic treatment. It may remineralize tooth structure because it forms a coating of calcium, phosphate, and silica-rich inorganic compounds that bonds to the enamel surface because when mixed with saliva, bioactive glass releases Ca2+, Na+, and PO_4_^3^ ions [13]. Lower shear bond strength (SBS) may lead to bracket debonding, which can be an inconvenience for both the dentist and patient [14]. The cost and time of therapy are increased as a result of this. It is crucial to find out whether the remineralizing chemicals can restore the mineral content of the demineralized enamel without compromising the SBS of the orthodontic brackets. A safe material should not reduce the bond strength below the minimum required for orthodontic brackets, which is between 6 and 8 MPa [14].

It is quite evident that there is a contrasting finding when the use of remineralizing agents is discussed in the previous literature. Furthermore, none of the aforementioned literature has produced any data that report the effects that remineralizing agents have on bracket bonding both before and after acid etching.

## Materials and methods

The research was conducted at the Department of Pediatric and Preventive Dentistry at the Government Dental College and Hospital in Nagpur, India. The Institutional Ethical Committee (approval no. IEC/03/03) gave its approval for this research. The extracted premolars were selected without any cracks or defects and stored in 10% formalin. All the selected teeth were divided equally into six subgroups depending on before and after acid etching and one separate control group for the in vitro study design. The sample size was estimated using the data obtained from a previous study conducted by Al-Kawari et al. [4] and t-tests (i.e., difference between two dependent means (matched pairs)). Eighty-four premolar teeth (12 teeth in each group x seven groups, including the control group). The selection criteria of the groups used in the study were divided into six subgroups and one separate control group.

The inclusion criteria include freshly extracted human premolar teeth, teeth with normal anatomy, and teeth from patients with no history of orthodontic treatment. The exclusion criteria include carious teeth, teeth with fractures and cracks, teeth with fluorosis, teeth with pulpal pathology, and teeth of patients who are smokers or tobacco chewers.

Procedure

After being cleaned, the extracted premolars were embedded in acrylic resin blocks prepared from molds measuring 25 x 20 mm using self-polymerizing resin. All teeth were cleaned with pumice and water for 15 seconds and then rinsed and dried for the same amount of time.

Experimental Groups

The experimental groups were divided as per selection criteria, and bracket bonding was done as follows:

Group-A1 (12) SDF before the acid-etching group: Enamel surfaces were pre-treated with SDF with the help of an applicator tip and left undisturbed for three minutes. The enamel was acid-etched for 20 seconds in 37% phosphoric acid, rinsed, and dried in accordance with the manufacturer's instructions. A bracket was held with a bracket-positioning tweezer, which was used to secure the bracket while the adhesive was loaded on the mesh surface of the bracket. When the bracket was properly positioned over the enamel, any excess adhesive material was removed with the other end of the bracket-placing tweezer. The bracket bonding procedure was completed using a curing light for 30 seconds.

Group-A2 (12) SDF after the acid-etching group: We followed the manufacturer's directions and acid-etched the enamel surface with 37% phosphoric acid for 20 seconds before cleaning and drying it. Using an applicator tip, SDF was applied to the enamel surfaces and then left alone for three minutes. A bracket-positioning tweezer was used to secure the bracket while the adhesive material was placed onto the mesh surface of the bracket. When the bracket was properly positioned over the enamel, any excess adhesive material was removed with the other end of the bracket-placing tweezer. The bracket-bonding procedure was completed using a curing light for 30 seconds [12].

Group-B1 (12) CPP-ACP before the acid-etching group: Casein-phosphopeptide amorphous calcium phosphate (CPP-ACP) paste was applied to enamel surfaces with the use of an applicator tip and left undisturbed for 30 minutes. The enamel was acid-etched for 20 seconds in 37% phosphoric acid, rinsed, and dried in accordance with the manufacturer's instructions. A bracket-positioning tweezer was used to secure the bracket while the adhesive was placed onto the mesh surface of the bracket. When the bracket was properly positioned over the enamel, any extra adhesive was removed with the other end of the bracket-placing tweezer. The bracket bonding procedure was completed using a curing light for 30 seconds.

Group-B2 (12) CPP-ACP after the acid-etching group: We followed the manufacturer's directions and acid-etched the enamel surface with 37% phosphoric acid for 20 seconds before cleaning and drying it. Casein-phosphopeptide amorphous calcium phosphate (CPP-ACP) paste was applied to enamel surfaces with the use of an applicator tip and allowed to sit undisturbed for 30 minutes. A bracket-positioning tweezer was used to secure the bracket while the adhesive was placed onto the mesh surface of the bracket. When the bracket was properly positioned over the enamel, any extra adhesive material was removed with the other end of the bracket-placing tweezer. The bracket bonding procedure was completed using a curing light for 30 seconds.

Group-C1 (12) NaF before the acid-etching group: The enamel surface was pre-treated with sodium fluoride varnish with the help of an applicator tip and left undisturbed for three minutes. The enamel was acid-etched for 20 seconds in 37% phosphoric acid, rinsed, and dried in accordance with the manufacturer's instructions. A bracket-positioning tweezer was used to secure the bracket while the adhesive was placed onto the mesh surface of the bracket. When the bracket was properly positioned over the enamel, any extra material was removed with the other end of the bracket-placing tweezer. The bracket bonding procedure was completed using a curing light for 30 seconds.

Group-C2 (12) NaF after the acid-etching group: We followed the manufacturer's directions and acid-etched the enamel surface with 37% phosphoric acid for 20 seconds before cleaning and drying it. Using the applicator tip, sodium fluoride varnish was applied to the enamel surfaces and left alone for three minutes. A bracket-positioning tweezer was used to secure the bracket while the adhesive was placed onto the mesh surface of the bracket. When the bracket was properly positioned over the enamel, any extra material was removed with the other end of the bracket-placing tweezer. The bracket bonding procedure was completed using a curing light for 30 seconds.

Control group: The enamel was acid-etched for 20 seconds in 37% phosphoric acid, rinsed, and dried in accordance with the manufacturer's instructions. A bracket-positioning tweezer was used to secure the bracket while the adhesive was placed onto the mesh surface of the bracket. When the bracket was properly positioned over the enamel, excess material was removed with the other end of the bracket-placing tweezer. After 30 seconds of exposure to curing light, the bonding of the brackets was complete.

**Figure 1:**
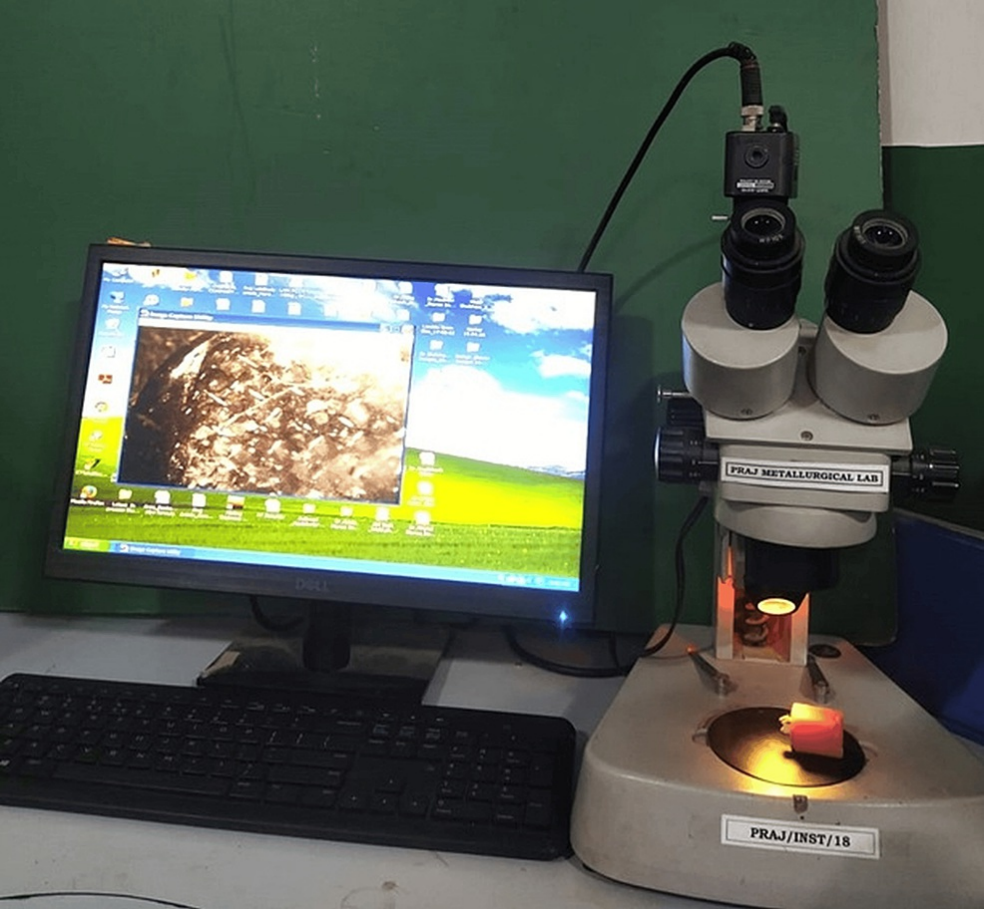
Stereomicroscope capturing images

**Figure 2:**
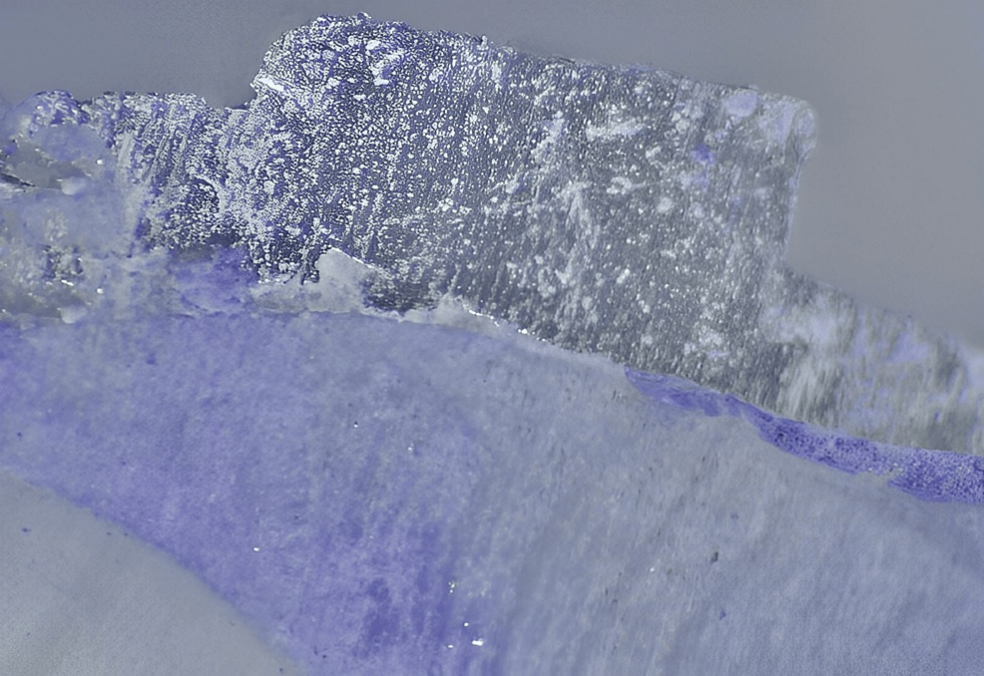
Stereomicroscopic image

All samples were thermocycled (500 cycles at 50 and 550 °C, dwell length 30 seconds) after 24 hours in distilled water at 370 °C after the completion of the bracket bonding procedure (model no. 50C: 051SA 550 C, Mahavir, LG, India). To perform the debonding test, a custom stand was used to hold each tooth steady while a computerized software-based universal testing machine applied a constant shear force with a knife-edged blade as close to the tooth/bracket interface as possible at a cross-head speed of 1 mm per minute. The shear bond strength was calculated as the force per unit area necessary to make the bracket loose. After all of the brackets were removed, the surfaces of the teeth were inspected using a stereomicroscope (model: XTL 3400 E, Wuzhou New Found Instrument Co., Ltd., China), and the images were processed using an image analysis system (model: MVIG 2005, Chroma System Pvt. Ltd., India) (Figures 1, 2).

Analysis

The comparison of the shear bond strength before and after acid etching within each group was conducted using independent t-tests. This analysis assessed the differences in the shear bond strength for each group separately. Meanwhile, the comparison of shear bond strength before acid etching and after acid etching between the four groups was performed using a one-way analysis of variance (ANOVA) test. This analysis examined the overall differences in shear bond strength among the different groups. All statistical tests and analyses were carried out using IBM SPSS Statistics for Windows, version 20 (released 2011; IBM Corp., Armonk, New York, United States). The significance level was set at p < 0.05, indicating that results with a p-value less than 0.05 were considered statistically significant.

## Results

The selection criteria of the groups used in the study are shown in Table 1.

**Table 1:** Selection criteria of the groups used in the study and divided into six subgroups and one separate control group. CPP-ACP, casein phosphopeptide amorphous calcium phosphates; SDF, silver diamine fluoride; NaF, sodium fluoride

Agents	Subgroups	Total number of samples
SDF	Before acid etching (Group A1)	12
	After acid etching (Group A2)	12
CPP-ACP	Before acid etching (Group B1)	12
	After acid etching (Group B2)	12
5% NaF	Before acid etching (Group C1)	12
	After acid etching (Group C2)	12
Acid etching + adhesive bracket bonding	Control group	12

The comparison of the shear bond strength of four different groups used before orthodontic bracket bonding on the basis of after acid etching and before acid etching, respectively. After acid etching, the SDF group saw a small but insignificant increase in shear bond strength. The CPP-ACP group saw a statistically significant improvement in shear bond strength after acid etching. After acid etching, the 5% NaF group had a statistically significant increase in shear bond strength. Shear bond strength was measured prior to acid etching, and the CPP-ACP group had the greatest value, followed by the control group and SDF group. The 5% NaF group had the weakest bonds, and the difference between the groups was statistically significant. The CPP-ACP group had the highest shear bond strength following acid etching, followed by the 5% NaF group (Table 2).

**Table 2:** Comparison of the shear bond strength before and after acid etching within each group Independent t-test; * indicates significant difference at p ≤ 0.05 CPP-ACP, casein phosphopeptide amorphous calcium phosphates; SDF, silver diamine fluoride; NaF, sodium fluoride

Groups	Acid etching	Mean value of bond strength (MPa)	SD	Mean difference	t-value	p-value
SDF	Before acid etching	7.48	0.65	-0.53	-1.620	0.119
	After acid etching	8.01	0.92			
CPP_ACP	Before acid etching	11.01	0.98	-2.08	-5.125	0.001*
	After acid etching	13.08	1.01			
5% NaF	Before acid etching	6.09	0.77	-3.22	-10.110	0.001*
	After acid etching	9.31	0.80			

The least bond strength was seen in the SDF group, and the difference between the three groups was significant, as shown in Table 3.

**Table 3:** Comparison of the shear bond strength before acid etching and after acid etching between four groups One-way ANOVA test; * indicates a significant difference at p ≤ 0.05. CPP-ACP, casein phosphopeptide amorphous calcium phosphates; SDF, silver diamine fluoride; NaF, sodium fluoride

Etching	Acid etching	Mean value of the bond strength (MPa)	SD	F value	p-value
Before	SDF	7.48	0.65	84.927	0.001*
	CPP-ACP	11.01	0.98		
	5% NaF	6.09	0.77		
	Control	10.83	1.20		
After	SDF	8.01	0.92	99.772	0.001*
	CPP-ACP	13.08	1.01		
	5% NaF	9.31	0.80		

## Discussion

An important clinical issue associated with orthodontic brackets is the demineralization of enamel. Plaque that has been allowed to accumulate on the affected surface for an extended period of time might cause WSLs to develop. This is a typical condition that arises as a direct result of insufficient attention to one's dental hygiene [14]. Nevertheless, the use of remineralizing agents during fixed orthodontic treatment might help limit the amount of decalcification that occurs. The resistance of enamel to acids generated by certain cariogenic bacteria has been demonstrated to increase as a part of the process by which fluoride lowers decalcification and caries. The formation of fluorapatite from fluoride deposits in hydroxyapatite is thought to affect the bond's stability [15]. It is uncertain if the qualities of these preventive interventions will have a positive, neutral, or negative influence on the bracket SBS, despite the fact that they have all been demonstrated to lower caries and WSL incidence.

In this study, we examined how three remineralizing agents, SDF, CPP-ACP, and 5% NaF, affected the SBS of orthodontic brackets and compared the results. Because preserving a healthy enamel surface after the removal of orthodontic brackets is one of the fundamental aims of orthodontic treatment, the application of remineralization has generated a significant amount of attention among clinicians. Hence, the significant benefits of remineralizing are reflected in the production of new products that are available on the market. The most preferred remineralizing agent is fluoride. Through the formation of less soluble fluorapatite, the sodium fluoride solution applied topically promotes remineralization by lowering apatite dissolution [16]. This substance affects the tooth surface by dispersing easily accessible ions into the enamel, where they form stronger crystals. In addition, it has been demonstrated that remineralizing enamel with synthetic apatite or hydroxyapatite is advantageous [16]. However, CPP-ACP, a novel protein produced from milk, may one day change the face of oral healthcare by facilitating tooth remineralization. CPP acts as a stabilizer for the ACP in the solution. By keeping the WSL's calcium gradients and phosphate concentration high, it promotes rapid enamel remineralization [17]. When administered topically, CPP-ACP has been shown to behave as a reservoir for calcium and phosphate, a buffer for free calcium, and a facilitator of remineralization [18].

Fluoride treatment prior to acid etching increases enamel resistance to acid, which might affect bracket adherence and reduce SBS values [19]. Fluoride-rich enamels typically require a longer etching time and are thought to be more resistant to acid etching [20]. In order to benefit from higher fluoride uptake without affecting the bond strength of adhesive resin, Hirce et al. [21] suggested the use of topical fluoride following acid etching. Tabrizi and Cakirer [22] recommended that CPP-ACP, a preventive agent prior to bracket bonding, should be used either alone or in conjunction with fluoride before acid etching. Moreover, Cehreli et al. [23], using self-etching systems, found that using CPP-ACPF before acid etching did not change bracket SBS, but using its non-fluoride version, CPP-ACP did reduce bracket SBS by a lot. Kecik et al. [24] have shown that acidulated phosphate fluoride (APF) or CPP-ACP considerably boosts the SBS of orthodontic brackets, whether they are used alone or together. Cossellu et al. [25] performed a comparative evaluation of six different types of remineralizing agents on the SBS of orthodontic brackets. The study concluded that CPP-ACP paste and ozone do not compromise the SBS of the brackets. However, fluoride, glycine, and hydroxyapatite decreased the SBS value. Baka et al. [26] examined the results of resin infiltration, micro-abrasion with 18% HCl and pumice, micro-abrasion without HCl, and CPP-ACP on demineralized enamel. Demineralized enamel has been shown to increase surface imperfections and reduce bond strength. The minerals were successfully restored with the aid of all the remineralizing agents tested. Resin infiltration or CPP-ACP can lower the SBS values, and micro-abrasive agents or a combination of both can lower the surface roughness. Meanwhile, Uysal et al. [27] demonstrated that CPP-ACP-treated samples had higher SBS than the demineralization group. However, neither the control nor the CPP-ACP-treated groups deviated much from the norm. As this would be in line with the results of the research, it is not unreasonable to assume that CPP-ACP was used to attain the enamel surface composition of the teeth in the control group. This difference across the study findings could be due to differences in materials used. Although the minimum acceptable SBS is not clear in the literature, Turk et al. [28] mentioned that the required SBS ranges from 13.0 to 21.0 MPa, while others suggested a range from 6.0 to 8.0 MPa [1]. It is important to remember that in vitro conditions do not closely resemble oral clinical outcomes, where there are noticeable large fluctuations in humidity, stress, temperature, and acidity. Reynolds previously reported that SBS values in the range of 5.9-7.8 MPa are sufficient for orthodontic applications [23]. We found that the SBS values obtained from the acid etching groups treated with SDF, CPP-ACP, and NaF varnish were all greater than the predicted range. Due to variances in techniques, including tooth selection, prevention agent selection, application time, fluoride concentrations, bonding technology, and thermal cycling, comparing the results of this research to those of others requires caution.

The limitations of the study are as follows. First, the present study was based on in vitro test results. However, within the scope of this study, when evaluating the data, it is important to keep in mind the limits of in vitro experiments. Second, while comparing the study results with the previous ones, the changes in methodology, such as the use of various types of preventive agents, application time, etching, and bonding systems, should be evaluated carefully. This in vitro study suggests that treating the enamel surface with protective agents before or after acid etching might be helpful without affecting the strength of the bond, within the limits of the study. These in vitro results would need to be backed up by more clinical studies that look at how well preventive treatments work before orthodontic brackets are bonded.

## Conclusions

The CPP-ACP group had the strongest shear bonds, followed by the control group and SDF group. There was a statistically significant difference between the groups, with the 5% NaF group having the weakest bonds. The CPP-ACP group had the highest shear bond strength following acid etching, followed by the 5% NaF group. The least bond strength was seen in the SDF group, and the difference between the three groups was significant. Based on these results, it can be concluded that the use of these remineralizing agents resulted in favorable values that did not have an effect on the SBS and were therefore safe to use with orthodontic brackets. When it comes to bonding orthodontic brackets, CPP-ACP pretreatment is superior to fluoride pretreatment in terms of effectiveness.
